# The influence of growth time on the identification of *Bartonella henselae* strains by MALDI-TOF mass spectrometry

**DOI:** 10.1590/S1678-9946202466009

**Published:** 2024-02-05

**Authors:** Karina de Almeida Lins, Cristiane Santos Cruz Piveta, Carlos Emilio Levy, Marina Rovani Drummond, Luciene Silva dos Santos, Alessandra Sussulini, Paulo Eduardo Neves Ferreira Velho

**Affiliations:** 1 Universidade Estadual de Campinas, Faculdade de Ciências Médicas, Departamento de Dermatologia, Campinas, São Paulo, Brazil; 2 Universidade Estadual de Campinas, Faculdade de Ciências Médicas, Departamento de Patologia Clínica Campinas, São Paulo, Brazil; 3 Universidade Estadual de Campinas, Instituto de Química, Departamento de Química Analítica, Campinas, São Paulo, Brazil

**Keywords:** Bartonella henselae, MALDI-TOF MS, Neglected infectious diseases

## Abstract

*Bartonella* spp. are bacteria responsible for neglected diseases worldwide. *Bartonella henselae* is the species most associated with human infections. It is associated with a large spectrum of clinical manifestations and is potentially fatal. The identification of *Bartonella* spp. is considered a challenge in clinical routine. These bacteria are fastidious, and the time required to isolate them varies from one to six weeks. MALDI-TOF mass spectrometry has emerged as an application for research on *Bartonella* spp. , and has still been little explored. We investigated whether three different *B. henselae* strains with different growth times—14 and 28 days—could be correctly identified by MALDI-TOF mass spectra fingerprint comparison and matching. We found that the spectra from strains with different growth times do not match each other, leading to misidentification. We suggest creating database entries with multiple spectra from strains with different growth times to increase the chances of accurate identification of *Bartonella* spp. by MALD-TOF MS.

## INTRODUCTION

*Bartonella* spp. are fastidious Gram-negative bacteria responsible for emerging and reemerging neglected infectious diseases worldwide^1^. Most *Bartonella* spp. are zoonotic and transmitted by blood-sucking arthropod vectors^2^. Infection by these bacteria is associated with a large spectrum of clinical manifestations, which mainly affect immunocompromised patients and people living in poor sanitation^1,3^.

Among *Bartonella* species associated with human diseases, *Bartonella henselae* is the most common. Infected individuals can develop cat scratch disease (CSD), a condition characterized by regional self-limiting lymphadenopathy, possibly accompanied by unspecific symptoms such as fever or fatigue^3^. Domestic cats are the main reservoirs of these bacteria, and transmission among cats has been proven to require fleas^4^. Cat contact is considered a risk factor, and transmission to humans is related to infected animal scratches or bites, mainly via inoculation of contaminated vector feces on the lesioned skin^5^. In addition to the classical symptoms, *B. henselae* infection is also associated with conditions such as prolonged fever of unknown origin, endocarditis, bacillary angiomatosis, bacillary peliosis, neuroretinitis, uveitis, encephalopathy, anemia, osteomyelitis, hepatitis, and others^6^.

*Bartonella* spp. detection is considered a challenge. The isolation of these slow-growing bacteria usually requires one to six weeks of incubation under specific conditions, and their low biochemical reactivity makes identification by phenotype-based methods unfeasible^7^. There is no single gold standard methodology for diagnosing *Bartonella* infection. Multistep platforms combining prolonged culture on enriched media, PCR amplification, and gene sequencing, have been used to improve the detection of *Bartonella* sp.^8^. Despite providing increased sensitivity and specificity, this approach is time-consuming, expensive, and laborious, and can yield false-negative results. There is a great need to develop faster and more effective approaches.

In this context, the advent of matrix-assisted laser desorption/ionization time of flight mass spectrometry (MALDI-TOF MS) has revolutionized the routine identification of microbial pathogens. It is progressively replacing current methods in clinical microbiology laboratories^9^.

The MALDI-TOF MS analysis is based on mass spectra comparison. A reference spectrum can be determined as a fingerprint characteristic of each microorganism and stored on a database. Bacterial identification is achieved when a spectrum obtained from an unknown bacterium matches with a reference one. It only requires a small number of isolated colonies and can be easily carried out immediately after bacterial growth. One of the main advantages of this method is the time saved for analysis: using conventional methods, it can take 24 to 48 h, but using MALDI-TOF MS, it takes less than one hour, which can be life-changing for critical patients^10^. This method is reliable, reproducible^11^, and cost-saving^12^. The potential of bacterial identification, including that of rare species, can be enhanced with more reliable database entries and software upgrades. Direct identification from positive blood culture samples, subspecies typing, and biomarkers for antimicrobial resistance discovery are some examples of other applications that have been explored^13^.

The identification of uncommon and difficult-to-culture bacteria such as *Bartonella* spp. is promising^14^, but the number of studies on this subject is still limited. The difficulty of isolating these microorganisms is one of the main obstacles. Nevertheless, one study successfully determined a unique reference spectrum for each of the 20 strains representing 17 *Bartonella* species and correctly identified 36 out of 39 blindly tested *Bartonella* sp. strains at the species level^15^. However, the incubation time required to isolate colonies was not considered in that study. Growth time can be a source of variation in bacterial mass spectra^16^, which can compromise spectral fingerprint-based identification. Although this does not significantly impact the analysis of common microorganisms that are easily isolated in routine clinical laboratory, it could particularly affect the identification of fastidious bacteria.

Here, in order to explore the potential use of MALDI-TOF MS for the identification of *Bartonella* spp., we created database entries for three different *B. henselae* strains with two different growth times—14 and 28 days. The accuracy of the spectral fingerprint match-based identification of newly cultured bacterial strains was then evaluated.

## MATERIALS AND METHODS

Three *B. henselae* strains recovered from three different cats in Campinas, Brazil, were provided by the Instituto Adolfo Lutz Microorganism Collection (World Data Centre for Microorganisms Registry Nº 282): Brazil-1 IAL 3714, Brazil-1 IAL 3715 and Brazil-1 IAL 3716. Their identities were previously confirmed by PCR amplification and 16S rRNA gene sequencing, as previously described^17^. The strains were cultured on solid media prepared with 6 g of Bordet-Gengou agar base in 117 ml of distilled water and 1.167 ml of glycerol. After sterilization and cooling to 50 °C, they were supplemented with 30% sheep blood. Incubation was carried out at 35 °C in a humidified, CO_2_-enriched environment for two different periods: 14 and 28 days.

MALDI-TOF MS measurements were performed using the Microflex MALDI-TOF MS system (Bruker Daltonics), which was calibrated daily with Bruker Bacterial Test Standard (BTS, Bruker). Considering the presence and absence of peaks as spectral fingerprints, reference spectra for each strain at each incubation time were acquired according to the manufacturer’s protocols and stored in a database. Lastly, the same bacterial strains were once again cultured and analyzed, and new spectra were acquired and compared with the previously obtained reference spectra.

For the acquisition of reference spectra, bacterial extraction was carried out by preparing a homogenous suspension of two or three isolated colonies harvested in 300 µL of sterile water. The suspension was precipitated with ethanol (900 µL) twice, and the resultant pellet was treated with a formic acid and acetonitrile mixture. The bacterial extract (1 µL) was spread on eight sample positions of the stainless steel target plate (Bruker) and left to dry at room temperature. The samples were then overlaid with 1 µL of a matrix solution composed of α-cyano-4-hydroxycinnamic acid (Sigma) saturated with 2.5% (v/v) trifluoroacetic acid and 50% (v/v) acetonitrile and subsequently allowed to cocrystallize.

FlexControl 3.0 software (Bruker) was used for spectral acquisition. The analysis was carried out in a *m/z* range of 2000-20000 Da in the positive linear mode. The spectra resulted from the sum of several laser shots over five different regions of the same sample. FlexAnalysis 2.4 software (Bruker) was used for visual inspection and quality control of the obtained spectra. Outlier and flatline spectra were rejected, and peaks of resolution > 500 were selected in the range of 3,000-10,000 Da, defined based on a previous study that identified peaks suitable for identifying and differentiating *Bartonella* species within this range^15^. A reference spectrum was then determined using at least 20 spectra obtained for each strain at each growth time.

Biotyper 3.0 software (Bruker) was used for spectral comparison of the analyzed bacterial strains with the references. Four technical replicates of the bacterial extract (as described above) were directly transferred to the target plate. Immediately after drying at ambient temperature, 1 µL of matrix was added and allowed to dry. Spectra were then acquired and analyzed. According to the manufacturer’s recommendations, species-level identification is achieved when the spectra of a tested bacterial strain show the best match with a reference spectrum in the database, with a score greater than 2.0. Scores ≥ 1.7 and < 2.0 were defined as reliable genus identification, and scores < 1.7 were defined as unreliable identification. Score values range from 1.0 to 3.0.

## RESULTS

Table 1 shows the average score values for comparison with the database entries of the spectra obtained from four replicates of each *B. henselae* strain and growth time. The columns represent the database entries, and the rows represent the spectra of the newly cultured bacterial strains. Overall, higher score values (> 2) were obtained when comparing *B. henselae* strains with the same growth time. However, when the spectra from the strains incubated for 14 days were compared with the database entries incubated for 28 days and vice versa, regardless of type, lower score values (< 2) were observed. This indicates that there was low similarity between the spectral fingerprints in the comparative analysis, suggesting spectral differences between strains of the same species with different growth times. Different bacterial strains with the same growth time were indistinguishable.

**Table 1:** **-** Score values obtained from Biotyper (Bruker) comparative analysis of three *B. henselae* strains (IAL 3714, IAL 3715 and IAL 3716) at two different growth times (14 and 28 days).

Newly cultured *B. henselae* strains	Database entries of *B.henselae* strains					
	IAL 3714 14 days	IAL 3715 14 days	IAL 3716 14 days	IAL 3714 28 days	IAL 3715 28 days	IAL 3716 28 days
**IAL 3714** **14 days**	2.515 2.551 2.529 2.441	2.276 2.073 2.163 2.150	2.145 2.202 2.247 2.132	1.886 1.857 1.783 1.655	1.556 1.602 1.508 1.170	1,489 1.731 1.589 1.085
**IAL 3715** **14 days**	2.090 2.017 2.037 1.956	2.678 2.688 2.624 2.546	2.287 2.406 2.221 2.386	1.440 1.397 1.147 1.072	1.747 1.651 1.476 1.037	1.839 1.658 1.225 1.372
**IAL 3716** **14 days**	2.072 2.147 2.237 2.127	2.288 2.369 2.379 2.365	2.705 2.738 2.680 2.682	1.358 1.477 1.671 1.134	1.433 1.233 1.346 1.462	1.473 1.616 1.169 1.593
**IAL 3714** **28 days**	1.825 1.992 1.905 1.892	1.093 1.736 1.572 1.674	0.456 1.572 1.408 1.357	2.390 2.527 2.451 2.359	1.923 2.041 2.186 2.026	2.004 2.082 2.128 2.092
**IAL 3715** **28 days**	1.579 1.821 1.862 1.909	1.384 1.801 1.573 1.354	1.109 1.711 1.644 1.597	1.809 2.063 2.137 2.230	2.018 2.346 2.088 2.265	2.065 2.273 2.362 2.548
**IAL 3716** **28 days**	1.414 1.569 1.581 1.334	0.835 0.838 1.170 0.503	1.354 1.234 0.758 0.641	2.068 2.033 1.956 1.998	2.137 2.185 2.177 2.014	2.231 2.491 2.225 2.226

## DISCUSSION

Although MALDI-TOF MS has proven to be a reliable and reproducible tool for bacterial identification, it is known that factors such as the growth time of the bacterial strains influence its reproducibility^16,18^. Growth time can particularly affect the identification of bacteria that are difficult to culture, such as *Bartonella* spp., for which the time required for isolation is variable. A study reporting a 5-year experience of culturing *Bartonella* from human samples reported that primary isolates could usually be obtained after 14 days, but prolonged incubation of up to 45 days was required in some cases^19^.

Using the same equipment and experimental procedure and considering the comparison score values from the Biotyper software, we found that the different *B. henselae* strains tested at the same growth time were indistinguishable (score values ≥ 2). However, the *B. henselae* strains at 14 days of incubation did not match the database entries at 28 days and vice versa, regardless of whether they were of the same type or not, indicating spectral differences. Longer incubation periods lead to the use of nutrients and the production of metabolic wastes that can chemically modify the media, requiring the bacteria to adapt to environmental changes^16^. Considering this, we hypothesize that spectral differences between strains with different growth times may be associated with ultrastructural changes in *B. henselae* over incubation periods, as could be observed in strains that were recovered after *in vivo* passage in mice^20^. These hypothetical ultrastructural and spectrometric alterations in fastidious bacteria may be related to the expression of highly ectopic proteins and could interfere with the development of diagnostic tests and vaccines.

Arnold *et al*.^16^ set out to use MALDI-TOF MS to monitor the growth of an *Escherichia coli* culture periodically from six to 84 h. They found that the mass spectra could vary qualitatively and quantitatively according to culture’s growth time and strongly recommended that experimental parameters, namely incubation time, be standardized to ensure reliable results. Our findings regarding spectral changes between *B. henselae* with different growth times corroborate this previous study, but we draw attention to the fact that, unlike *E. coli* and other easily cultured bacteria, growth time cannot be accurately controlled for fastidious microorganisms such as *Bartonella* spp.. Review studies^10^ report that the reliability and accuracy of microorganism identification by MALDI-TOF MS strongly depends on the number of database entries. Therefore, we suggest that database entries from different growth times of *Bartonella* spp. strains be considered to avoid misidentification of these fastidious bacteria.

In addition to the potential spectral variations associated with the time required to isolate *Bartonella* spp., the absence of reference spectra of these bacteria in commercial databases can also compromise their identification in clinical routine. Fournier *et al*.^15^ previously obtained MALDI-TOF MS reference spectra for several *Bartonella* species. However, the reference spectra for *Bartonella* spp. were not available in the commercial database provided by manufacturers until the date of this study, limiting their identification in nonspecialized laboratories. Croxatto *et al*.^18^ reported that studies evaluating the performance of routine identification by MALDI-TOF MS show that misidentification is mainly associated with the absence or mislabeling of reference spectra in the database. Based on these facts, the inclusion of reference spectra for uncommon and/or neglected bacteria such as *Bartonella* spp. on available commercial databases could improve research and diagnosis of these pathogens and should be considered by manufacturers.

## CONCLUSION

In conclusion, growth time has proven to be a source of variation in *B. henselae* mass spectra fingerprints, which may pose a concern about how the growth time of strains can compromise the identification of *Bartonella* sp. by MALDI-TOF MS. Database entries from *Bartonella* spp. strains with different growth times should be considered to avoid misidentification.
